# Effects of Fish Oil Supplementation on Oxidative Stress Biomarkers and Liver Damage in Hypercholesterolemic Rats

**DOI:** 10.3390/nu14030426

**Published:** 2022-01-18

**Authors:** Joana Érica Lima Rocha, Mariely Mendes Furtado, Renato Sampaio Mello Neto, Ana Victória da Silva Mendes, Ana Karolinne da Silva Brito, José Otávio Carvalho Sena de Almeida, Emerson Iuri Rodrigues Queiroz, José Vinícius de Sousa França, Maísa Guimarães Silva Primo, Ana Lina de Carvalho Cunha Sales, Andreanne Gomes Vasconcelos, Wanessa Felix Cabral, Selma Aparecida Souza Kückelhaus, José Roberto de Souza de Almeida Leite, Ana Karina Marques Fortes Lustosa, Massimo Lucarini, Alessandra Durazzo, Daniel Dias Rufino Arcanjo, Maria do Carmo de Carvalho e Martins

**Affiliations:** 1 Department of Biophysics and Physiology, Federal University of Piauí, Teresina 64049-550, Brazil; ericarocha6@hotmail.com (J.É.L.R.); marielymf@live.com (M.M.F.); renato.sampaio.mn@gmail.com (R.S.M.N.); victoriams18@hotmail.com (A.V.d.S.M.); anakarolinnesb@hotmail.com (A.K.d.S.B.); otavios.almeida@hotmail.com (J.O.C.S.d.A.); emersoniuri@ufpi.edu.br (E.I.R.Q.); vinicius.sfranca@ufpi.edu.br (J.V.d.S.F.); maisaguimaraessp@gmail.com (M.G.S.P.); ana.lina123@gmail.com (A.L.d.C.C.S.); daniel.arcanjo@ufpi.edu.br (D.D.R.A.); 2 Research Center in Morphology and Applied Immunology, Faculty of Medicine, University of Brasília, Brasília 70910-900, Brazil; andreannegv@gmail.com (A.G.V.); wanessa.felix@unb.br (W.F.C.); selmask@gmail.com (S.A.S.K.); jrsaleite@gmail.com (J.R.d.S.d.A.L.); 3 Galeno Farmácia de Manipulação, Virgínia Regina Fortes Castelo Branco e Cia Ltda, Teresina 64001-260, Brazil; ana_lustosa@uol.com.br; 4 CREA-Research Centre for Food and Nutrition, Via Ardeatina 546, 00178 Rome, Italy; massimo.lucarini@crea.gov.it (M.L.); alessandra.durazzo@crea.gov.it (A.D.)

**Keywords:** dyslipidemias, oxidative stress, antioxidants, fish oil, dietary supplements

## Abstract

Metabolic syndrome, especially its component related to dyslipidemia, is related to the development of nonalcoholic fatty liver disease (NAFLD), which is a disease with a significant global prevalence. Supplementation with omega-3 polyunsaturated fatty acids emerged as a complementary therapeutic possibility for dyslipidemia, but its benefits are questioned. This paper aims at evaluating the effects of fish oil supplementation in rats with hypercholesterolemia induced by hypercholesterolemic diet (HD). The study design is based on an experimental model in which the animals were randomly divided into 3 groups: G1 (standard commercial feed + saline solution); G2 (hypercholesterolemic diet + saline solution) and G3 (hypercholesterolemic diet + fish oil) over a period of 16 weeks. Metabolic control parameters and oxidative stress biomarkers were evaluated according to standardized methodologies. The G3 group showed significantly lower values of plasma concentrations of TG, and hepatic myeloperoxidase as well as higher erythrocyte superoxide dismutase activity (*p* < 0.05). Regarding histopathological analysis, there was lipid accumulation in the liver of animals from group G2; meanwhile, hepatocytes reorganization and expressive reduction of lipid vacuoles and hepatic TG content was observed in group G3. This study demonstrated how fish oil supplementation reduced the plasma concentration and hepatic content of triglycerides, as well as liver tissue damage in histopathological analysis.

## 1. Introduction

Dyslipidemias are disorders of lipoprotein metabolism circulating in the blood, characterized by high serum concentrations of total cholesterol (TC), triglycerides (TG), low-density lipoprotein-linked cholesterol (LDLc), and/or low high-density lipoprotein-linked cholesterol (HDLc) [1]. Metabolic syndrome (MS) includes aspects of dyslipidemia, in addition to abdominal obesity, high blood pressure, and insulin resistance. These factors are related to a state of proinflammatory and oxidative stress and a dysregulation of the innate immune system. Therefore, MS is considered an important condition associated with the development and maintenance of noncommunicable chronic diseases [2]. The increase in the circulating concentration of lipids is accompanied by metabolic alterations and deleterious effects, including hepatic damage [3]. Several strategies are adopted for prevention or treatment of these harmful effects caused by dyslipidemia, among which are nutritional interventions with partial replacement of saturated fatty acids in the diet by mono and polyunsaturated fatty acids [4].

The nonalcoholic fatty liver disease (NAFLD) is related to the presence of hepatic steatosis (intrahepatic fat of at least 5% of liver weight) when alcohol consumption and other secondary causes are not significantly present, and the aforementioned risk factors are markedly involved [5]. In addition, other factors may contribute to its development or evolution, including genetics, endocrine disorders, autoimmune diseases, and use of drugs [6,7]. The prevalence of NAFLD is 23% in those with type 2 diabetes mellitus, 42% in patients with metabolic syndrome, and 69% in those with dyslipidemias [8].

The development of NAFLD occurs due to multiple factors related to the metabolism of free fatty acids (FFA) and their intermediates. One of the main factors involved is the oxidation of FFA, when they are not packaged in TGs or VLDLc, which can directly lead to the production of reactive oxygen species (ROS), normally limited by the antioxidant system. However, the antioxidant system may be ineffective in maintaining ROS at physiological levels when FFAs are produced in excess during NAFLD progression. Oxidative stress can lead to hepatocellular damage through lipid peroxidation, which can directly activate cell necrosis and activation of the Fas ligand apoptotic pathway. Therefore, according to Manne, Handa, and Kowdley [9], “…excessive accumulation of TGs in the liver may be associated with toxicity because these TGs can still be hydrolyzed to FFA and overload the liver’s ability to metabolize them and the accumulation of free cholesterol in the liver. Liver is also lipotoxic and occurs due to unrestricted uptake of circulating low-density lipoprotein hepatic rescue receptor”.

The supplementation with omega-3 polyunsaturated fatty acids was proposed as a therapeutic intervention for the reduction of plasma triglyceride concentration by decreasing hepatic lipogenesis and by increasing lipoprotein lipase (LPL) activity, accelerating the catabolism of VLDL and chylomicrons [10,11,12]. There are three fatty acids in the omega 3 series: alpha linolenic acid (ALA), eicosapentaenoic acid (EPA), and docosahexaenoic acid (DHA). The ALA is present in some cereals, legumes, and vegetable oils, while the fatty acids EPA and DHA are present in greater amounts in fish from deeper waters, like salmon and sardines [13,14]. The use of omega-3 fatty acids is mainly recommended as an auxiliary resource in the therapy of hypertriglyceridemia. However, the recommendation of use of supplementation with fish oil capsules in individuals with or without atherosclerotic disease, within the primary prevention of cardiovascular events is still being questioned [15,16,17].

In this context, omega-3 fatty acids may be able to reduce TG concentrations, oxidative stress, and the degree of hepatic steatosis [18,19,20]. Given the above, this study evaluated the effect of treatment with fish oil (EPA+DHA), an important source of omega-3 fatty acids, on biomarkers of lipid profile, liver function and oxidative stress in induced dyslipidemic Wistar rats fed with a hypercholesterolemic diet.

## 2. Materials and Methods

### 2.1. Ethical Aspects

The animals were obtained from the Central Animal House from Federal University of Piauí (Teresina, Brazil) and maintained during all experimental protocols in collective polypropylene cages (4 animals/box), in a room at controlled temperature of 22 ± 2 °C, relative humidity of 40 ± 5%, and light-dark cycle of 12 h, with free access to food and water. All experimental protocols were submitted and approved by the Ethics Committee on Animal experimentation (CEUA) of the Federal University of Piaui (approval No. 446/18). The procedures regarding the euthanasia of animals were in accordance with Resolution No. 1000 of 11 May 2012, of the Federal Council of Veterinary Medicine.

### 2.2. Experimental Protocol

Forty-eight male Wistar rats (*Rattus norvergicus*), 7 to 8 months old, weighing (375 ± 29 g) were used. The hypercholesterolemic diet used in this study was based on the methodology by Baracho et al. [21], and consisted of the addition of 0.1% cholesterol, 0.5% cholic acid and egg yolk as a source of cholesterol (4 units/100 g of standard commercial feed—Presence^®^ Nutrição Animal, Paulínia, SP, Brazil). The animals were randomly divided into 3 groups: G1 (standard commercial feed + saline solution 1 mL/day PO); G2 (hypercholesterolemic diet + saline solution 1 mL/day PO) and G3 (hypercholesterolemic diet + 1 mL/day of fish oil PO).

**Figure 1:**
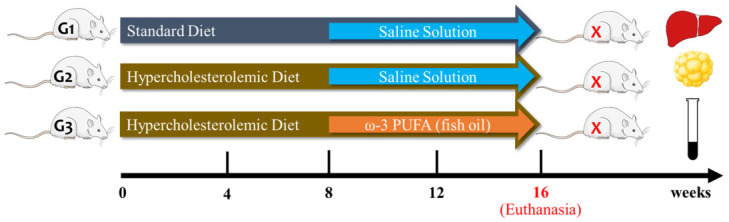
Experimental design of induction of hypercholesterolemia and fish oil supplementation in male Wistar rats.

After two weeks of acclimatization with standard commercial feed, animals of G2 and G3 groups received exclusively the hypercholesterolemic diet, and G1 received only standard commercial feed, during the entire study. Then, after 8 weeks of respective diets, both G1 and G2 groups were orally treated with saline solution (negative control), and G3 group was orally treated with fish oil (1 mL/day) (Figure 1).

In humans, the recommendation for supplementation with fish oil is approximately 1–3 g/day to obtain benefits against cardiovascular diseases [10,11,12,22]. In this study, 1 mL of fish oil (equivalent to 310 mg of omega-3 fatty acids) per animal was daily administered for 8 weeks. Considering the straight range of body weight of the rats used in our experimental protocols, the differences among volumes to be administrated would be very low. Therefore, in this study we decided to use the content of 1 fish oil capsule (1 mL) per animal. The experimental protocol was repeated 3 times to allow us to assess reproducibility. This average daily consumption in rats, when submitted to a dose conversion for humans based on body surface area [23], is in accordance with recommendations for beneficial supplementation against cardiovascular diseases according to recommendations of the American Heart Association [10], Brazilian Society of Cardiology [11] and European Society of Cardiology [12].

### 2.3. Fish Oil Gel Capsules and Fatty Acids Composition

The fish oil was obtained commercially in gel capsules, and its fatty acids (FAs) content analyzed by GC-MS comprises 33.57% of saturated FAs, 30.28% of monounsaturated FAs, 31.1% of n-3 PUFAs, and 3.61% of n-6 PUFAs (data not shown). The n-6/n-3 ratio was 0.116. This composition agrees with most of fish oil capsules available at market. Moreover, a study of Nevigato et al. [24] assessed the fatty acids content of different fish oil capsules, and content ranges of PUFAs are similar to the fish oil used in this study.

### 2.4. Determination of the Centesimal Chemical Composition of the Feed

The moisture content and ash, lipids, protein, and carbohydrate contents were determined according to recommendation of the Association of Official Analytical Chemists (AOAC) [10] in the standard commercial feed given to the control group (G1) and in the hypercholesterolemic diet, given to the groups G2 and G3. Total energy values were estimated by means of Atwater’s energy conversion factors: 4 kcal/g for proteins, 4 kcal/g for carbohydrates and 9 kcal/g for lipids.

The analysis of the centesimal composition showed that the hypercholesterolemic (HD) diet had significantly higher amounts of energy and lipids compared to that of the standard commercial feed (*p* < 0.05). The commercial feed showed higher values (*p* < 0.05) with respect to the values of carbohydrates and proteins. The moisture content was higher in the HC diet when compared to that of the standard commercial feed (Table 1).

### 2.5. Euthanasia, Organ Harvesting and Weighing

Twenty-four hours after the treatment period, fasted animals were anesthetized by intraperitoneal administration of lidocaine 10 mg/kg and thiopental sodium 50 mg/kg. Venous blood was collected and centrifuged at 3000 rpm for 15 min at 4 °C using the centrifuge Centribio/Daiki 80-2B for plasma determination of glucose, lipid profile, biomarkers of oxidative stress in plasma including malondialdehyde (MDA) and myeloperoxidase (MPO); albumin, total protein, aspartate aminotransferase (AST), and alanine aminotransferase (ALT). Besides, erythrocytes were washed three times with 5 mL of 0.9% saline solution followed by centrifugation for determination of superoxide dismutase (SOD) activity in erythrocytes. After blood collection, the animals were euthanized by intraperitoneal administration of lidocaine 10 mg/kg and thiopental sodium 150 mg/kg. Thereafter, liver samples were also collected for determination of MDA and activity of the antioxidant enzymes Catalase (CAT) and SOD, as well as the content of nonprotein sulfhydryl groups (SH-NP), lipid extraction, and histopathological analysis.

### 2.6. Determination of Glycemia and Lipid Profile

Plasma concentrations of glucose, TC, HDL-c and TG were determined by colorimetric enzymatic method in a Labmax Plenno^®^ biochemical analyzer using Labtest^®^ kits according manufacturer instructions (Labtest Inc. Lagoa Santa, MG, Brazil). Non-HDL-c was calculated by the difference between total cholesterol and HDL-c [12]. All concentrations were expressed in mg/dL.

### 2.7. Determination of Albumin, Total Proteins, and Enzymes of Hepatic Cytolysis

Plasma concentrations of albumin, total proteins, and alanine aminotransferase (ALT) and aspartate aminotransferase (AST) activity were determined by colorimetric enzymatic method in a Labmax Plenno^®^ biochemical analyzer using Labtest^®^ kits according manufacturer instructions (Labtest Inc. Lagoa Santa, MG, Brazil).

### 2.8. Determination of Plasma and Hepatic Malondialdehyde (MDA) Concentrations

MDA concentrations were determined by the production of thiobarbituric acid reactive substances (TBARS) [25]. Therefore, 200 µL of plasma or liver homogenate were added to 350 µL of 20% acetic acid (pH 3.5) and 600 µL of 0.5% thiobarbituric acid. The mixture was incubated in a water bath for 45 min at 100 °C and subsequently cooled in an ice bath for 15 min. Subsequently, 50 µL of 8.1% sodium dodecyl sulfate (SDS) was added and the mixture was centrifuged for 15 min at 12,000 rpm at 25 °C.

The supernatant was read in a spectrophotometer at the wavelengths of 532, 510, and 560 nm; and the corrected absorbance was calculated using the formula proposed by Pyles; Stejskal; Einzig [26]. A calibration analytical curve was prepared using MDA as standard. The results were expressed as nmol of MDA per mL of plasma or per g of liver homogenate (nmol/g liver).

### 2.9. Determination of Plasma and Hepatic Myeloperoxidase (MPO) Activity

Measurement of MPO activity was performed based on the oxidation rate of the substrate o-dianisidine in the presence of H_2_O_2_, which is determined by the change in absorbance measured at 450 nm [27]. The reading was performed in an ELISA microplate with 10 µL of plasma or homogenate and 200 µL of the reading solution, consisting of distilled H_2_O, phosphate buffer pH 6.0, 1% H_2_O_2_ and 5 mg of o-dianisidine. Two readings were performed at the absorbance of 450 nm at 1-min intervals. The results were expressed as units of MPO/µL of sample (U/µL).

### 2.10. Determination of Erythrocyte and Hepatic Superoxide Dismutase (SOD) Activity

The evaluation of erythrocyte SOD was performed based on the amount of SOD capable of inhibiting 50% of nitrite formation. 100 µL of the sample (lysate or homogenate) were added to 1110 µL of sodium phosphate buffer pH 7.4, 75 µL of 20 mM L-methionine, 40 µL of 1% Triton X-100, 75 µL of 10 mM hydroxylamine chloride, 100 µL of 50 µM EDTA. Subsequently, the mixtures were incubated in a water bath at 37 °C for 10 min, immediately after this process riboflavin (80 µL) was added and exposed to light for 10 min [28].

Absorbance measurements was performed in an Elisa SynergyMx plate with 50 µL of the reaction medium and 50 µL of Griess reagent at wavelength of 543 nm and the erythrocyte SOD was expressed in U/g Hb. The measurements were performed in duplicate and the results were expressed in U/mg of protein for hepatic SOD.

### 2.11. Determination of Hepatic Catalase (CAT) Activity

CAT activity was determined based on quantification of the rate of decomposition of hydrogen peroxide (H_2_O_2_) by CAT with decreased optical density at 230 nm and 37 °C [28]. The reaction medium was prepared using 9 mL of 10 nM H_2_O_2_, 0.5 mL of 5 mM Tris HCl 1 M EDTA buffer pH8.0, and 0.4 mL of Milli-Q water. For the reaction, 7.5 µL of the homogenate diluted in potassium phosphate buffer and 250 µL of the reaction medium were mixed. The samples were incubated at 37 °C and absorbance readings were taken every minute for 6 min in a SynergyMx automated plate reader at 230 nm. The results were expressed as U/mg of protein. Total protein concentration was determined using a commercial colorimetric kit according to the manufacturer’s instructions (Labtest Diagnóstica S.A., Lagoa Santa, MG, Brazil). One unit (U) of catalase corresponded to the enzyme activity that allowed the hydrolysis of 1 µmol of H_2_O_2_ per minute at 37 °C in pH 8.0 [29].

### 2.12. Determination of Hepatic Concentrations of Nonprotein Sulfhydryl Groups (SH-NP)

For the determination of SH-NP content, each liver sample (300 mg) was homogenized in 3 mL of ice-cold 0.02 M EDTA. Subsequently, 2 mL of the homogenate was added to 2 mL of 10% trichloroacetic acid (TCA). After centrifugation at 3000 rpm for 15 min, 2 mL of 0.4 M Tris EDTA 0.2 M pH 8.9 was added. Henceforth, 50 µL of 5,5′-dithio-bis (2-nitrobenzoic acid) (DTBN) was added to the sample and absorbance measurements were read at wavelength 412 nm [30].

### 2.13. Histopathological Analysis of Liver Tissue

After fixation in 10% buffered formalin and transferred to 70% alcohol, the material was processed using Mod 808 histotechnical equipment (ANCAP Equipamentos Eletroeletrônicos LTDA, São Paulo, Brazil) by dehydration in increasing concentrations of ethanol (80%, 90%, 95% and absolute), followed by diaphanization in xylene. After impregnation and paraffin embedding, the fragments were sectioned having a 5 μm thickness and stained with Hematoxylin and Eosin (HE). The slides were analyzed at 40x objective using a ScanScope histological slides scanner (Aperio) (Leica Biosystem Inc., Newcastle, DE, USA) blinded in all histological sections. Aspects of general tissue architecture were evaluated qualitatively, in the whole slide without considering artifacts, using the Aperio ImageScope software version 12.4.0.5043.

### 2.14. Hepatic Triglyceride and Total Cholesterol Content

Liver (100 mg) was homogenized with 700 µL NaCl (1 M) and, subsequently, 2 mL of chloroform/methanol (2:1) were added. After centrifugation at 4000 rpm for 5 min, 3 phases were formed, and the lower (methanolic) phase was removed for subsequent drying in a boiling water bath. The sample was resuspended in 500 µL of Triton X100/Methanol (2:1) followed by vortex agitation. Immediately after, TG and TC levels were determined by enzymatic colorimetric assay [30,31].

### 2.15. Statistical Analysis

This is a study with data not paired with randomized groups. All data were submitted to the Shapiro–Wilk normality test and had their numerical parameters of normality evaluated before being considered as parametric data. The results were expressed as mean and standard error of the mean. For comparison of means between groups, an analysis of variance (ANOVA) was performed, followed by Tukey’s test for multiple comparison, considering a confidence level of 95% (*p* < 0.05). Graphs and statistical analyses were performed using GraphPad Prism statistical software version 7.00 for Windows (GraphPad Software, La Jolla, CA, USA, www.graphpad.com; accessed on 22 November 2021).

## 3. Results

The effect of fish oil supplementation was described on following parameters: (i) lipid profile and glycemia; (ii) biochemical markers of liver function and on macroscopic and histological aspects; (iii) biomarkers of lipid peroxidation; (iv) biomarkers of antioxidant activity; and (v) hepatic cholesterol and triglyceride concentrations.

### 3.1. Effect of Fish Oil Supplementation on Lipid Profile and Glycemia in Animals with Hypercholesterolemia

**Figure 2:**
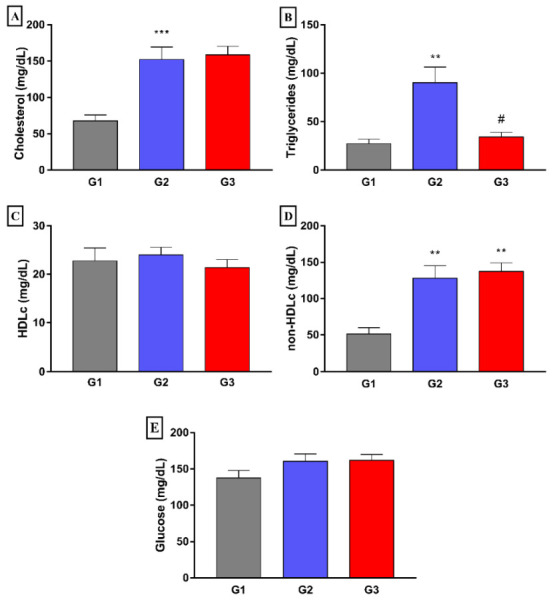
Effect of 8 weeks fish oil supplementation (1 mL/day) on plasma concentrations of cholesterol (**A**), triglycerides (**B**), HDLc (**C**), non-HDLc (**D**), and glucose (**E**). G1 = standard commercial feed + saline solution; G2 = hypercholesterolemic diet + saline solution; G3 = hypercholesterolemic diet + fish oil. ** *p* < 0.01 and *** *p* < 0.001 when compared to G1. # *p* < 0.05 when compared to G2.

The group treated with fish oil (G3) showed significantly lower mean plasma triglyceride concentrations (*p* < 0.05) when compared to that of the G2 group. However, there were no differences between the groups regarding total cholesterol, HDL-c, and non-HDL-cholesterol (Figure 2). Besides, no differences were also found in plasma glucose concentrations between the different experimental groups.

### 3.2. Effect of Fish Oil Supplementation on Biochemical Markers of Liver Function and on Macroscopic and Histological Aspects of the Liver of Hypercholesterolemic Animals

**Figure 3:**
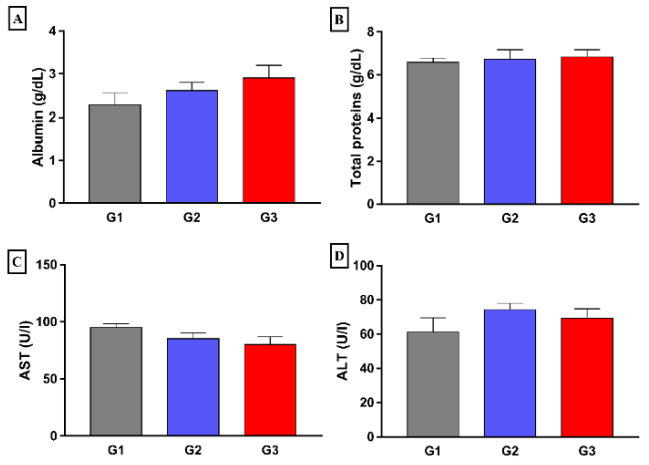
Effect of 8 weeks fish oil supplementation (1 mL/day) on biochemical markers of liver function (**A**, Albumin; **B**, Total protein; **C**, ALT; and **D**, AST). G1 = standard commercial feed + saline; G2 = hypercholesterolemic diet + saline; G3 = hypercholesterolemic diet + fish oil; AST = Aspartate aminotransferase; ALT = Alanine aminotransferase.

**Figure 4:**
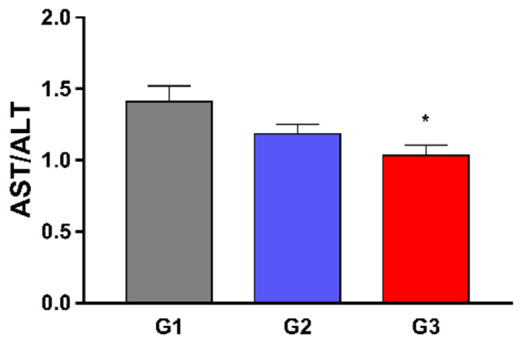
Effect of 8 weeks fish oil supplementation (1 mL/day) on De Ritis Index. Rite index, AST/ALT- Aspartate aminotransferase -AST, Alanine aminotransferase—ALT. G1 = standard commercial feed + saline; G2 = hypercholesterolemic diet + saline; G3 = hypercholesterolemic diet+ fish oil. * *p* < 0.05 when compared to G1.

No statistically significant differences were found between the groups regarding biochemical markers of liver function albumin, total protein, AST, and ALT, (Figure 3). Besides, a slight significant decrease was observed in the DeRitis index after treatment of hypercholesterolemic rats with fish oil (G3) when compared to that of hypercholesterolemic control (G2) (Figure 4).

**Figure 5:**
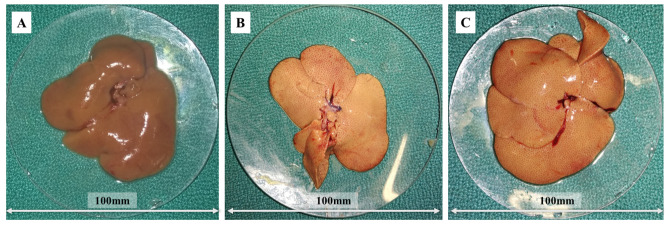
Effect of fish oil supplementation (1 mL/day) for 8 weeks on liver gross pathology: (**A**) G1: standard diet + 1 mL of saline solution; (**B**) G2: hypercholesterolemic diet + 1 mL of saline solution; (**C**) G3: hypercholesterolemic diet + 1 mL of fish oil.

Regarding morphological analysis, we observed changes in the coloration of the organs of animals in group G2, which received HD and saline solution, when compared to those that received the standard diet (G1). The pale and yellowish coloration suggests accumulation of lipids, characterizing hepatic steatosis in these animals. However, in the animals of the group treated with fish oil (G3), less evident changes were observed when compared to that of G2 group (Figure 5).

**Figure 6:**
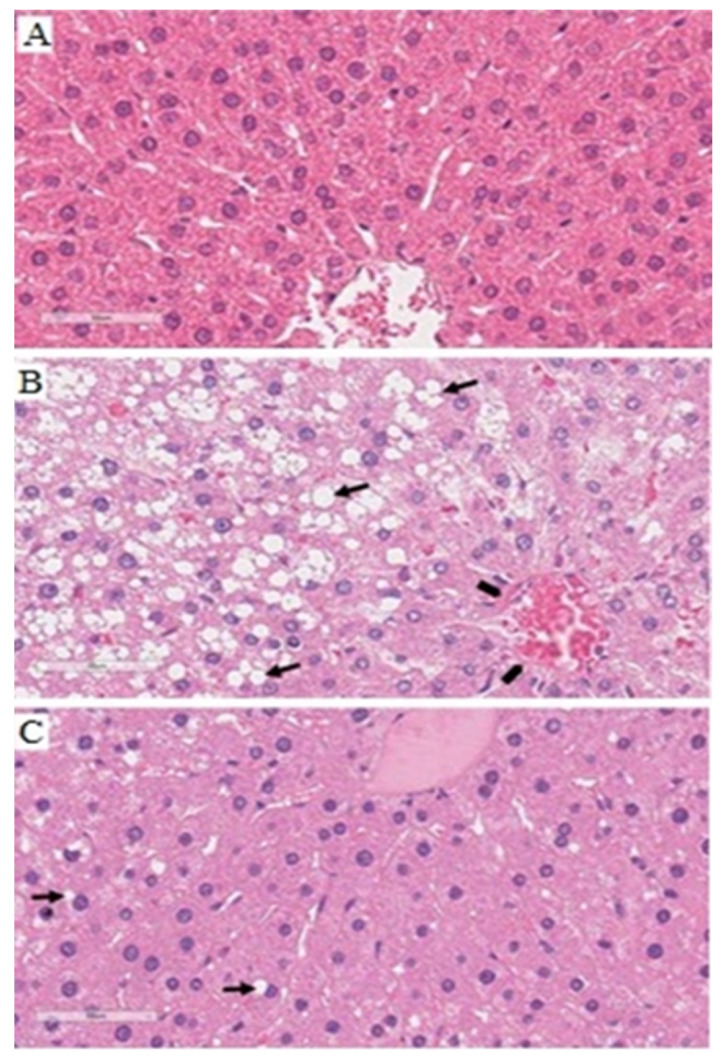
Effect of 8 weeks fish oil supplementation (1 mL/day) on histological aspects of liver of animals of groups G1 (**A**), G2 (**B**), and G3 (**C**). Representative photomicrographs of histological sections of rat liver from groups G1 (**A**), G2 (**B**) and G3 (**C**). Hematoxylin-eosin (HE) staining, 400x magnification. Scale bar: 50 µm. In B and C, short arrow shows lipid macrovesicle; in B, pentagon shows fibrosis on wall of midlobular vein.

Histological analysis of the liver of animals in the G1 group revealed normal lobular architecture, with hepatic cords arranged typically near the central-lobular vein, besides hepatocytes with acidophilic cytoplasm and characteristic fine granular appearance, with abundant mitochondria; central, large, spherical nucleus, and scattered chromatin with one or two evident nucleoli (Figure 6A). On the other hand, in the dyslipidemia group (G2) a large extension of clear, vacuolized lobular areas was observed, possibly indicating severe hepatic impairment. Also, numerous lipid macrovesicles were observed (short arrow), besides cytoplasmic degeneration and thin fibrosis on the central-lobular vein wall (pentagon) (Figure 6B).

Interestingly, in the group supplemented with fish oil (G3), the parenchyma showed hepatocytes typically arranged in strands, with lower vacuole content compared to the G2 group. Some macrovesicles suggestive of lipid accumulation in the cytoplasm of hepatocytes are indicated by the short arrow (Figure 6C).

### 3.3. Effect of Fish Oil Supplementation on Biomarkers of Lipid Peroxidation in Animals with Hypercholesterolemia

**Figure 7:**
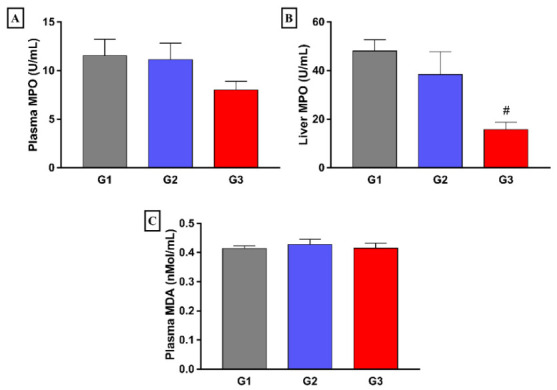
Effect of 8 weeks fish oil supplementation (1 mL/day) on plasma MPO (**A**), hepatic MPO (**B**) and plasma MDA (**C**) concentrations. G1 = standard commercial feed + saline solution; G2 = hypercholesterolemic diet + saline solution; G3 = hypercholesterolemic diet + fish oil; MPO = myeloperoxidase; MDA = malondialdehyde; # *p* < 0.05 when compared to G2.

Regarding plasma MDA and MPO, no significant differences were observed between the experimental groups. On the other hand, significantly lower hepatic MPO values were found in the group treated with fish oil (G3) compared to that of the group fed with HD and treated with saline (G2). Furthermore, HD did not alter the concentration of MPO in the group that received the standard diet (Figure 7).

### 3.4. Effect of Fish Oil Supplementation on Markers of Antioxidant Activity in Animals with Hypercholesterolemia

**Figure 8:**
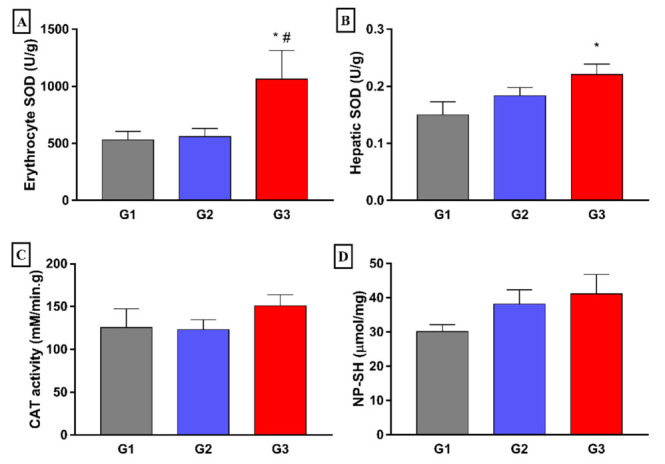
Effect of 8 weeks fish oil supplementation (1 mL/day) on erythrocyte (**A**) and hepatic (**B**) SOD activities, CAT activity (**C**) and NP-SH concentration (**D**) in liver tissue. G1 = standard commercial feed + saline solution; G2 = hypercholesterolemic diet + saline solution; G3 = hypercholesterolemic diet + fish oil; SOD = superoxide dismutase; NP-SH = non-protein sulfhydryl; CAT = catalase. * *p* < 0.05 when compared to G1. # *p* < 0.05 when compared to G2.

When analyzing antioxidant activity markers, it was observed that the animals in the G3 group exhibited significantly higher values of erythrocyte SOD activity in relation to the G2 group (*p* < 0.05) (Figure 8A). In the hepatic tissue, no statistically significant differences were found between the groups regarding SOD and catalase activity, nor in relation to the hepatic content of non-protein sulfhydryl groups (NP-SH).

### 3.5. Effect of Fish Oil Supplementation on Hepatic Cholesterol and Triglyceride Concentrations in Hypercholesterolemic Animals

**Figure 9:**
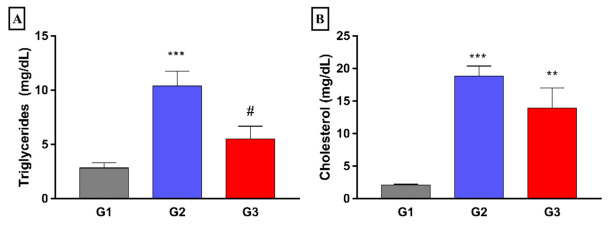
Effect of 8 weeks fish oil supplementation (1 mL/day) on hepatic triglycerides (**A**) and cholesterol (**B**) concentrations. G1 = standard commercial feed + saline solution; G2 = hypercholesterolemic diet + saline solution; G3 = hypercholesterolemic diet + fish oil; ** *p* < 0,005 and *** *p* < 0.001 when compared to G1. # *p* < 0.05 when compared to G2.

Concerning the content of lipids in the liver, it was observed that the G2 group displayed significantly higher levels of triglycerides and cholesterol (*p* < 0.001) when compared to that of the G1 group. When analyzing the G3 group, it was found that the levels of TG were significantly lower (*p* < 0.05) when compared with that of G2, although no difference was found in liver cholesterol content (Figure 9).

## 4. Discussion

The findings in the present study indicate protective effect of fish oil supplementation in this dyslipidemia model as demonstrated by the reduction of plasma concentration and hepatic content of triglycerides, besides improvement in hepatic steatosis, observed in histopathological analysis, decrease in hepatic concentration of MPO, and increase in erythrocyte SOD concentration.

Fish oil supplementation reduced plasma and liver triglyceride concentrations in our study. According to the study by Godea et al. [32], where oral supplementation with fish oil in Wistar rats aged 65 weeks and receiving or not receiving a high-fat diet, a beneficial effect of fish oil was reported. The reduction in TG serum concentrations with fish oil supplementation can be explained by decreasing hepatic lipogenesis and suppressing the expression of sterol regulatory element-binding protein-1c. As a result, there is decreased expression of cholesterol, fatty acid, and TG synthesis enzymes [33]. In addition, fish oil is able to increase β-oxidation of fatty acids, resulting in a reduction in the substrate available for TG synthesis [27]. About the reduction of hepatic TG, a similar result was reported by Haimeur et al. [34] with the supplementation of 0.5% fish oil in the high-fat diet compared to rats receiving HD alone for 8 weeks, and they attributed these results to increased beta oxidation and decreased lipogenesis induced by high-fat diet.

In this study, supplementation with fish oil caused no change in plasma glucose concentrations in the groups studied. Similar result was found by Yuan et al. [35] with male rats that 8–9 weeks; weight—180–200 g. In the study by Yuan et al. [35], rats were fed with standard control or HD diet and a HD diet containing fish oil for 16 weeks, in which the HD diet induced severe macrovesicular steatosis and inflammatory infiltration and the fish oil treated group developed mild hepatic steatosis. Similar results were found in the present study in which hepatic steatosis was also demonstrated in the HD group and lipid accumulation in the fish oil treated group.

Serum levels of total protein and albumin were similar between groups. As albumin and other proteins are produced by the liver, changes in these parameters may indicate liver fibrosis, but they are not used alone, but together with other parameters in different tests and scores. For example, albumin is part of the NAFLD Fibrosis Score along with age, fasting glucose, BMI, platelets, and AST/ALT, which has a sensitivity of 43% and a specificity of 96% for detecting liver fibrosis; Haptoglobin, ApoA1 and a2-macroglobulin are some of the components of Fibrotest (sensitivity: 92%, specificity: 71%); a2-macroglobulin is part of the Hepascore (sensitivity: 75%, specificity: 84%); and albumin and globulin together with other clinical and laboratory parameters achieved 57% sensitivity and 90% specificity in an adult model with NAFLD and diabetes. The hypothesis of liver fibrosis was ruled out as our results did not show changes in albumin and globulins [36].

We did not find any statistical difference when evaluating AST and ALT separately, but when evaluating the ratio between them we found a lower value in the group fed with high fat content and treated with fish oil. The release of ALT and AST from liver cells into the bloodstream represents hepatocellular damage or death. The absence of statistical difference between the groups can mean that there was not enough hepatocellular damage to change the levels of transaminases [37,38]. The ratio of serum AST and ALT activities was first described by Fernando De Ritis in 1957 and has since been known as the “De Ritis ratio”. The clinical application of this index is wide and can be misinterpreted. In general, a higher De Ritis index indicates liver disease. Both transaminases, mainly ALT, can be elevated due to overweight, obesity and metabolic syndrome. For this reason, in cases of hepatic steatosis, the De Ritis index is usually lower than 1. When there is steatohepatitis, the index is usually higher than 1. However, there is the possibility of an altered De Ritis index in healthy patients and a normal index in sick patients due to individual physiological variations [38]. De Ritis index reduced in the fish oil supplemented group can signify the presence of hepatic steatosis. The untreated dyslipidemic group showed no change in the index. However, this does not exclude the possibility of diagnosis, as previously explained.

Macroscopic and microscopic analysis of the livers of the studied animals demonstrated that fish oil supplementation reduced the progression of NAFLD. There are two classic hypotheses that try to explain the progression from initial NAFLD to more advanced conditions: the “two-hit” hypothesis and the modified “multiple hits” hypothesis. Both argue that it all starts with the accumulation of TG in the liver, therefore, if this first event is avoided, the evolution of the disease is also avoided. The study by Khadge et al. used male Sprague–Dawley rats induced to dyslipidemia by a high-fat Western diet alone or combined with fish oil for 16 weeks and showed that fish oil supplementation significantly improved dyslipidemia induced by Western diet, transaminase elevation, hepatic steatosis, inflammatory infiltration, and fibrosis. Furthermore, lower levels of proinflammatory genes (Mcp1, Socs2, Sema4a and Cd44) were identified in the group fed with fish oil [37].

No difference was found in plasma MDA levels between the hypercholesterolemic and control groups. When reactive oxygen species interact with PUFAs, lipid peroxidation occurs, resulting in the generation of MDA and other metabolites [39]. The systematic review with meta-analysis by Heshmati et al. [40] on the effects of omega-3 fatty acid supplementation on serum MDA levels in humans found that n-3 PUFAs have the potential to reduce the plasma MDA level in young and elderly adults, regardless of monthly dose was greater or less than 2000 mg or if supplementation was greater than or less than 10 weeks, but no difference was found in middle-aged adults. Even with a dose of 310 mg/day and supplementation for 8 weeks, we did not find results. This can be explained by a possible good functioning of the endogenous antioxidant system that counteracted the effects of the disease in all dyslipidemic animals, regardless of the treatment.

Regarding MPO activity, the group treated with fish oil showed lower plasma and hepatic concentrations of MPO compared to that of the HD group treated without fish oil supplementation. The MPO plays a role in microbial activity and human defense against pathogens by its participation in phagocytosis. Activated MPO releases substances such as hypochlorous acid and tyrosyl radical and when they react with hydrogen peroxide, they contribute to the occurrence of oxidative damage in cell membranes. Reduction in MPO concentrations is able to reduce inflammation and oxidative stress [41].

The group treated with fish oil for 8 weeks showed higher erythrocyte SOD activity when compared to the normal control group and hypercholesterolemia group (CH). Different results were described in the study by Drouin et al. [42] between control, EPA, and DHA groups with rats supplemented with 1% of EPA and DHA, respectively, in the diet of animals for 6 weeks. Richard et al. [43] reported that omega-3 may act indirectly as an antioxidant, reducing ROS production by eliminating superoxide.

The plasma bioavailability of n-3 PUFAs represents an important factor for their biological effects and benefits. In this sense, Ahn et al. [44] reported a sustained increase of both EPA and DHA for 8 h after consumption of 1 mL of fish oil per rat, followed by a decrease up to 12 h and a sustained concentration to 24 h. Besides, Martorell et al. [45] reported a marked increase in plasma DHA concentration in athletes supplemented with DHA for 8 weeks, and interestingly, this concentration is further increased after physical exercise. In this context, although plasma bioavailability of n-3 PUFAs was not carried out in the present study, previous reports documented this assessment after supplementation with fish oil obtained commercially. Furthermore, the control assessment of the gel capsules by GC-MS for identification and determination of fatty acids content supports the suitable quality of the fish oil in this study and reinforces the strength of the present findings.

## 5. Conclusions

This study aimed to evaluate the effects of fish oil rich in omega-3 fatty acids (EPA and DHA) on serum and liver biomarkers of oxidative stress, lipid profile, and liver function in dyslipidemic individuals. We found that fish oil supplementation of dyslipidemic rats decreased serum and liver triglyceride levels, reduced the progression of NAFLD, decreased the hepatic MPO activity, and increased both erythrocyte and liver SOD activities. Therefore, treatment with fish oil had a protective effect against hypercholesterolemic diet-induced hepatic steatosis in rats, attributed to the improvement in lipid metabolism demonstrated by the reduction in plasma concentration and hepatic triglyceride content, as well as damage to liver tissue. These findings add to the current understanding of the benefits of n-3 PUFAs against NAFLD. Further research in this direction into the molecular mechanisms underlying these effects is widely open. Our limitations were a small n that made it difficult to find significant results in some tests and the absence of a predetermined protocol for liver macroscopy analysis.

## Figures and Tables

**Table 1:** Centesimal composition and calories (Kcal) of standard commercial feed and hypercholesterolemic feed.

Components	Standard Feed Mean ± SD	HC diet Mean ± SD	* *p* Value
Carbohydrates (%)	40.64 ± 0.83	38.53 ± 0.35	0.016
Proteins (%)	39.53 ± 0.97	28.16 ± 0.01	0.002
Lipids (%)	2.12 ± 0.08	16.08 ± 0.06	0.000
Humidity (%)	8.46 ± 0.16	9.65 ± 0.21	0.002
Ash (%)	9.24 ± 0.29	7.55 ± 0.08	0.001
VET (kcal)	339.79 ± 7.97	411.59 ± 0.88	0.000

SD = standard deviation; HC = hypercholesterolemic; VET = total energy value; * Student’s *t* test for independent samples (*p* < 0.05). Analysis performed in triplicate.
